# Occult presentation of spinal epidural abscess

**DOI:** 10.1002/pmrj.70025

**Published:** 2025-09-15

**Authors:** Rotem Hass, Vincenzo Pizzuti, Ashley Petrone, Brett Begley

**Affiliations:** ^1^ Department of Physical Medicine and Rehabilitation University of Nebraska Medical Center Omaha Nebraska USA; ^2^ Department of Medicine University of Colorado Boulder Colorado USA; ^3^ Department of Pathology, Anatomy, and Laboratory Medicine West Virginia University Morgantown West Virginia USA; ^4^ Department of Internal Medicine University of Nebraska Medical Center Omaha Nebraska USA

Spinal epidural abscesses (SEAs) commonly arise from hematogenous dissemination, typically occurring from direct extension from adjacent tissue or inoculation from procedures involving the spinal canal.^1^, ^2^ The incidence of SEA is uncommon, with 5.1 of 10,000 admissions annually being due to SEAs; however, the nonspecific syndrome of fever, back pain, and/or neurologic deficits are not always present, leading to failure of early detection and treatment.^3^ SEA can be a secondary complication, commonly the setting of bacteremia or osteomyelitis, with intravenous drug use, diabetes, and trauma as high‐risk factors, with *Staphylococcus aureus* being the most common pathogen.^4^, ^5^ In this letter, we describe the case of an 87‐year‐old man with an occult presentation of SEA precipitated by a urologic procedure and highlight important clinical management considerations (Figures 1 and 2).

**FIGURE 1:**
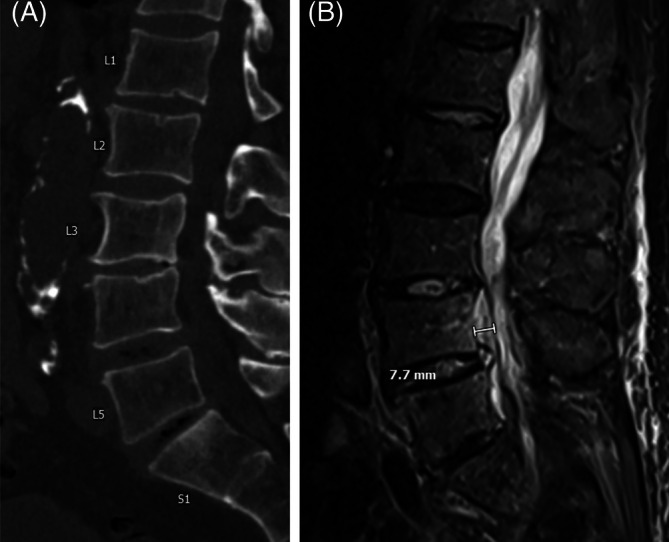
(A) Sagittal computed tomography of the lumbar spine without contrast demonstrating no visible epidural collection. (B) Sagittal magnetic resonance imaging of the lumbar spine performed 1 week later revealing a 7.7‐mm epidural abscess over L4–L5 vertebral bodies.

**FIGURE 2:**
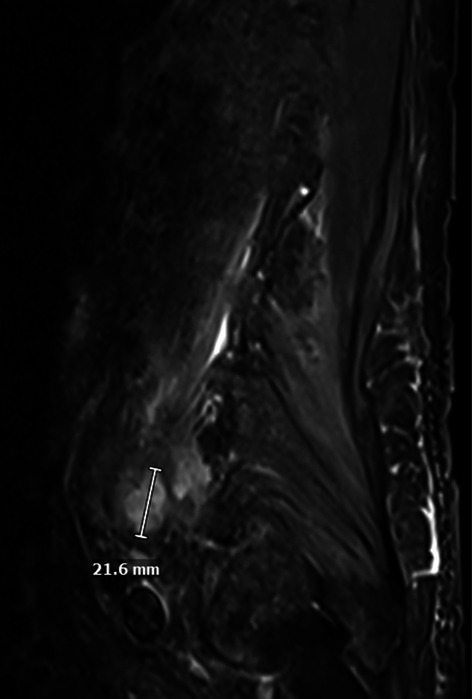
Sagittal magnetic resonance imaging view of left psoas abscess measuring 21.6 mm.

An 87‐year‐old man with a medical history of chronic back pain presented with worsening back pain over 2 weeks with no recent trauma or inciting events. Notably, he underwent a cystoscopy 3 weeks prior, experiencing a fever the following day prompting his initial presentation to the emergency department (ED). Given his recent urologic procedure and symptoms, a urinalysis was obtained, which showed pyuria. He was discharged on cephalexin pending urine culture results. Urine cultures later grew *Streptococcus* species.

He later presented the following week with persistent back pain with new onset of left lower extremity weakness, exacerbated by ambulation. Evaluation in the ED showed a normal complete blood count and basic metabolic panel, with an elevated CRP (c‐reactive protein) of 20 mg/dL. Given the new neurologic deficit, noncontrast computed tomography of the lumbar spine was performed showing degenerative changes throughout lumbar spine with severe foraminal stenosis, but no acute fractures, lesions, or abscesses were identified. The patient was discharged home with oral pain medications and a neurosurgery follow‐up for further evaluation as no infectious etiology was identified.

One week later he represented again with worsening pain and weakness. Labs showed an unremarkable complete blood count and basic metabolic panel, with the exception of an elevated erythrocyte sedimentation rate of 138 mm/h. The patient was admitted for inadequate pain control and to expedite neurosurgery's recommendation for lumbar magnetic resonance imaging that revealed a left psoas myositis with abscess and L4‐L5 discitis osteomyelitis with an SEA. Blood cultures, M protein, alpha/beta, and gamma globulin light chains were normal. Infectious disease and orthopedics recommended image‐guided biopsy of the psoas abscess that grew *Streptococcus gallolyticus pasteuranius*. The patient began a 6‐week course of intravenous ceftriaxone and was discharged with interval outpatient imaging and follow‐up.

Although lower back pain is common in older adults, the differential diagnosis for worsening chronic symptoms should remain broad. This patient's initial presentation lacked the classic triad of SEA, requiring a high index of suspicion to consider the diagnosis. Initial labs were also nonspecific, showing only pyuria and elevated inflammatory markers. Notably, there was no leukocytosis. This case was atypical in two key ways: the absence of leukocytosis (seen in 60%–80% of cases) and negative blood cultures (present in at least 60%).^2^, ^6^

For this patient, although the definitive etiology remains unclear, a urologic origin is likely. Omission of prophylactic antibiotics during cystoscopy may have permitted bacterial seeding of the psoas muscle, leading to abscess formation. Other common risk factors for SEA, including intravenous drug use and valvular vegetations on transthoracic echocardiogram, were absent. Given his recent urologic procedure, pyuria, and back pain, an earlier evaluation for pyelonephritis with computed tomography of the abdomen/pelvis may have identified the abscess sooner. On re‐presentation, the presence of neurologic deficits and elevated inflammatory markers should have prompted spinal imaging sooner. The absence of fever may reflect an age‐related blunted inflammatory response, underscoring the need for vigilance in older patients.

SEA is rare but can result in significant morbidity and mortality if not diagnosed and treated promptly. Although SEA often presents with back pain, fever, neurologic deficits, leukocytosis, and elevated inflammatory markers, these features may be absent. In this case, the patient had elevated inflammatory markers but no leukocytosis. Acute changes in patients' pain and new neurological deficits should indicate the need for advanced imaging if prior imaging was unremarkable and cause for the change is uncertain. Surgical intervention is often required in patients with neurologic deficits, alongside an extended course of intravenous antibiotics.

## CONSENT

Written informed consent was obtained from the patient for publication of this case report and any accompanying images.

## DISCLOSURE

None.
